# Metabolic syndrome and breast cancer risk

**DOI:** 10.1186/s43046-023-00203-1

**Published:** 2023-12-21

**Authors:** Amira M. Mohammed, Hosney B. Hamed, Maisa K. Noaman, Nelly Alieldin

**Affiliations:** 1 Department of Biostatistics and Cancer Epidemiology, South Egypt Cancer Institute, Assiut University, Assiut, Egypt; 2 Department of Clinical Pathology, South Egypt Cancer Institute, Assiut University, Assiut, Egypt; 3 Department of Biostatistics and Cancer Epidemiology, National Cancer Institute, Cairo University, Fom Elkhalig Square, Cairo, Egypt

**Keywords:** Breast cancer, Metabolic syndrome, Case–control

## Abstract

**Background:**

Limited data are available on metabolic syndrome and its relation to breast cancer risk in Egypt. We aimed to study metabolic syndrome and its individual components as risk of breast cancer.

**Methods:**

This case–control study recruited 112 breast cancer cases and 112 age-matched controls from Assiut University. In addition to demographic, clinical, and anthropoemetric characteristics, blood samples were collected from both study groups to evaluate metabolic syndrome and its individual components.

**Results:**

Mean age of breast cancer cases and control groups was 46.10 ± 4.34 and 45.66 ± 4.68 years, respectively. According to Joint Interim Statement (JIS) criteria for clinical diagnosis of metabolic syndrome, the overall prevalence of metabolic syndrome in all participants was 42.9%, and prevalence in breast cancer cases and control group was 57.14% and 28.6%, respectively, OR 33.33, 95% CI (1.91–5.81). BMI was more likely to be higher in breast cancer patients with a linear trend, *p* < 0.001. For individual components of metabolic syndrome, breast cancer cases were more likely to have high fasting blood glucose level, systolic and/or diastolic blood pressure, high triglycerides level, and low HDL-C as compared to the control group.

**Conclusion:**

Metabolic syndrome and its components were found to be associated with the risk of breast cancer. We believe that prevention or reversal of metabolic syndrome by raising community awareness for lifestyle changes could be an effective way in minimizing the toll of the disease.

## Introduction

Breast cancer is a leading cause of cancer mortality and the most common form of cancer affecting women worldwide and in Egypt [1]. Although there are different risk factors for breast cancer development, such as age, genetic factors, menstrual status, reproductive, and lifestyle, however, oncologists have not given the full attention to certain metabolic disorders which may have a major role in breast cancer development [2].

Syndrome X, insulin resistance syndrome, or Reaven syndrome are synonyms of metabolic syndrome (MS) which is a group of at least three of the following five medical conditions: central obesity, high blood pressure, high fasting blood sugar, high serum triglycerides, and low serum high-density lipoprotein (HDL) [3]. Metabolic syndrome threatens public health in the modern world, and its prevalence has increased worldwide [4], and the two main forces that are responsible for increasing the prevalence of this syndrome are the change in eating habits associated with the increase in high calorie-low fiber fast food consumption and the decrease in physical activity due to sedentary lifestyle and mechanized transportations. There is still little awareness of metabolic syndrome, which remains underdiagnosed, insufficiently treated, and unsuccessfully controlled [5]. If it is not handled well, MS is significantly associated with an increased risk of developing diabetes and cardiovascular complications [6]. In addition, patients with MS are more likely to develop cancer, increase the recurrence, and have a worse long-term prognosis. It has been identified as a possible risk factor for pancreatic, breast, prostate, and colorectal cancers [7]. In spite of the advanced strategies of treatment in the recent era, the mortality because of invasive breast cancer is still high. However, giving the attention to the primary prevention of breast cancer by targeting the metabolic factors remains to be determined, because the relationship between the metabolic factors and the pathogenesis of breast cancer is still not yet proven [8].

Recent studies found that metabolic syndrome and its components have an impact on the initiation, progression, response to treatment, and prognosis of breast cancer [9]. However, little is known about the prevalence of metabolic syndrome and its components among those initially diagnosed with breast cancer in Egypt; moreover, we aimed at evaluating the relation between metabolic syndrome and breast cancer risk.

## Methods

A case–control study was conducted at South Egypt Cancer Institute, SECI, and approved by the ethical committee at SECI, Assiut University (SECI-IRB, IORG0006563, approval number: 443). The study conformed to all requirements as governed by the Declaration of Helsinki. The present study included 224 females, 112 newly diagnosed pathologically confirmed breast cancer patients and 112 age-matched apparently healthy females as a control group. It was conducted from January 1st, 2020 to December 31st, 2021. Patients were consecutively recruited from the outpatient clinics of surgery in SECI. Eligible participants were adult females, newly diagnosed and confirmed breast cancer, mentally competent to answer the questionnaire, and willing to accept participating in the study, and control group were identified as visitors attending different departments of the institute during the same period of cases recruitment. They were matched to the cases on age ± 2 years. Relatives of patients with breast, ovarian, cervical, and colorectal cancer were excluded. Sample size was estimated upon a case–control study by Wu et al. (2018) [2], who found that prevalence of MS among breast cancer cases and controls was 32.6% and 18.2% respectively with an OR, 2.173 (95% CI, 1.793 to 2.633). Using EpiR (https://shiny.vet.unimelb.edu.au/epi/sample.size.mccs/) for sample size calculation of matched case–control studies with 1:1 ratio of cases and controls, 0.05 level of significance, and 90% power of test, it was decided to recruit a total 224 participants, 112 per group. Out of 239 approached females, 224 agreed to participate with a refusal rate of 6.3%.

### Material and tools of the study

An informed consent was taken from all participants after discussing with them the aim and methods of the study. A questionnaire was developed to collect relevant data through personal interview with face-to-face approach and from medical records of the cases. Data collected included demographics, medical history, and metabolic indicators of both study groups and pathologic diagnosis of breast cancer cases. Blood pressure and anthropometrics including waist circumference (cm), height (cm), and body weight (kg) were measured for each participant. Blood samples were collected from all participants for assessment of serum lipid level, fasting blood glucose level, and fasting insulin resistance. Blood tests were performed by the Department of Clinical Pathology at SECI, Assiut Iniversity.

### Definition of metabolic syndrome and its individual components

According to the Joint Interim Statement (JIS) criteria for the clinical diagnosis of metabolic syndrome in adults [10], patients were considered positive for the presence of metabolic syndrome if they had three or more of the following factors:

1. Elevated fasting blood glucose (FBG) ≥ 7.0 mmol/L (or drug treatment for elevated glucose).
2. Elevated blood pressure (systolic blood pressure (SBP) ≥ 130 mmHg and/or diastolic blood pressure (DBP) ≥ 85 mmHg) (or drug treatment for hypertension).
3. Elevated serum triglyceride (TG) ≥ 1.7 mmol/L (or drug treatment for elevated TG).
4. Reduced high-density lipoprotein (HDL-C) (1.0 mmol/L in male and 1.3 mmol/L in female) (or drug treatment for reduced HDL-C).
5. Elevated waist circumference (WC) (according to population and country-specific definitions). It is > 40 inches in males and 35 inches in females, according to the National Cholesterol Education Program Adult Treatment Panel III (NCEP ATP III) definition [11].

BMI was calculated as weight in kilograms divided by the square of height in meters. BMI measurements were categorized as:

1. BMI levels, < 18.5 kg/m^2^ = Underweight
2. 18.5–24.9 kg/m.^2^ = “Normal weight”
3. 25–29.9 kg/m.^2^ = “Overweight”
4. 30–39.9 kg/m.^2^ = Obese”
5. < 40 kg/m.^2^ = “Morbid obese” [12]

### Statistical analysis

SPSS package (IBM Corp. Released 2017. IBM SPSS Statistics for Windows, Version 23.0. Armonk, NY: IBM Corp) was used for data management and analysis. Chi-square *X*^2^ and/or Fisher Exact tests were used for comparing independent categorical variables and for testing of linear trends. Student’s *T*-test compared the means of two groups, and the Mann–Whitney test when data were not normally distributed. Logistic regression analysis was done to depict the independent risk factors of breast cancer. Adjusted odds ratio (OR), 95% confidence interval calculated likely of risk with different factors. It was done using forward likelihood ratio method, and covariates and factors included in models were those significant on univariate analysis, the 5 components of MS, BMI, age as a potential confounder, and analysis stratified by menopausal status as a control of confounding. *P*-value < 0.05 was set for the significant results.

## Results

### Characteristics of the study groups

A total of 224 participants, 112 breast cancer patients, and 112 age-matched females constituted the control group. Sociodemographic characteristics and risk factors of breast cancer of the two study groups are shown in Table 1. No significant difference was found between groups for most sociodemographic characteristics. The mean age for cases and control groups was 46.10 ± 4.34 and 45.66 ± 4.68 years, respectively. Most of the breast cancer cases and control group were married, 56.3% of cases and 48.2% of controls were illiterate, and only 5.4% in cases and 6.3% of controls had secondary school education or higher. Around 90% of each study group were housewives. Table 1 also shows higher frequencies of positive family history of breast cancer, diabetes mellitus, and hypertension among cases than control group, *p* = 0.001, 0.009, and 0.002, respectively.

**Table 1:** Sociodemographic characteristics and risk factors of breastcancer in breast cancer cases and control group

**Characteristics**	Breast cancer *N* = 112 No. (%)	Control *N* = 112 No. (%)	Total *N* = 224 No. (%)	*p*-value *
Age, years, mean ± SD (range)	46.10 ± 4.34 (40–67)	45.66 ± 4.68 (39–65)	45.89 ± 4.51 (39–67)	0.487
**Marital status**				
Single/divorced/widow	13 (11.6)	6 (5.4)	19 (6.6)	0.09
Married	99 (88.4)	106 (94.6)	205 (91.5)	
**Education**				
Does not read or write	63 (56.3)	54 (48.2)	117 (52.2)	0.296
Read and write	23 (20.5)	35 (31.3)	58 (25.9)	
< secondary school	20 (17.9)	16 (14.3)	36 (16.1)	
Secondary or higher	6 (5.4)	7 (6.3)	1 (5.8)	
**Occupation**				
An employee	8 (7.1)	12 (10.7)	20 (8.9)	0.349
House wife	104 (92.9)	100 (89.3)	204 (91.1)	
**Income/month**				
≤ 3000 Egyptian pound	61 (54.5)	51 45.5)	112 (49.9)	0.414
> 3000 Egyptian pound	49 (43.8)	58 (51.8)	107 (47.8)	
Refused to answer	2 (1.8)	3 (2.7)	5 (2.2)	
**Number living in the same house (mean ± SD)**	5.63 ± 1.73	5.42 ± 1.85	5.52 ± 1.79	0.389
**Family history of breast cancer**	29 (25.9)	11 (9.8)	40 (17.9)	0.001*
**Age at menarche (mean ± SD)**	12.15 ± 1.02	11.69 ± 1.07	12.06 ± 1.05	0.088
**Age at first child birth**	20.82 ± 2.83	20.46 ± 2.5	20.63 ± 2.72	0.508
**Full term pregnancies**	4.53 ± 1.5	4.64 ± 1.5	4.59 ± 1.5	0.456
**Breast feeding**	104 (92.9)	107 (95.5)	211 (94.2)	0.391
**Postmenopausal women**	30 (26.8)	28 (25.0)	58 (25.9)	0.760
**History of OCP/HRT**	48 (42.9)	41 (36.6)	89 (38.7)	0.494
**Comorbidities**				
Diabetes	24 (21.4)	10 (8.9)	34 (15.2)	0.009*
Hypertension	27 (24.1)	10 (8.9)	37 (16.5)	0.002*

^*^p-value is significant ≤ 0.05, *OCP* oral contraceptive pills, *HRT* hormonal replacement therapy

### Metabolic and anthropometric profile in the two study groups

Table 2 shows that breast cancer cases had significantly higher levels of total cholesterol, triglycerides, fasting blood glucose, weight, BMI, and systolic blood pressure, as well as a significantly lower level of HDL than control group. There is no significant difference regarding diastolic blood pressure, fasting insulin resistance, and waist circumference between the two groups. For BMI classification, breast cancer patients were more likely to be overweight, obese, or morbid obese 38.4%, 38.4%, and 7.1%, respectively, and control group were more likely to be normal or overweight, 34.5% and 37.5%, respectively (*p*-value for linear trend = 0.002).

**Table 2:** Metabolic and anthropometric profile of breast cancer cases and control groups

Characteristics	Breast cancer *N* = 112 No. (%)	Control *N* = 112 No. (%)	Total *N* = 224 No. (%)	*p*-value *
**Metabolic profiles (mean ± SD)**				
Total cholesterol (mg/dl)	212.55 ± 42.1	192.3 ± 40.04	202.4 ± 42.2	< 0.001
HDL-c (mg/dl)	40.01 ± 8.3	47.6 ± 11.4	44.3 ± 10.5	< 0.001
TG (mg/dl)	214.6 ± 103.6	151.6 ± 78.4	183.1 ± 96.9	< 0.00
FBG (mg/dl)	120.8 ± 60.9	95.8 ± 28.9	108.3 ± 49.1	< 0.001
FIR	0.368 ± 0.236	0.355 ± 0.193	0.361 ± 0.215	0.936
SBP	120.9 ± 7.5	118.4 ± 7.4	119.7 ± 7.5	0.012
DBP	80.4 ± 5.97	79.9 ± 4.3	80.5 ± 5.2	0.482
**Anthropometric profiles (mean ± SD)**				
Weight (kg)	77.6 ± 14.9	71.2 ± 13.5	74.4 ± 14.5	< 0.001
Waist circumference (cm)	90.5 ± 18.0	88.2 ± 15.1	89.3 ± 16.6	0.289
BMI (kg/m2)	29.7 ± 5.7	26.8 ± 4.8	28.3 ± 5.5	< 0.001
**BMI classification (kg/m2)**				
Underweight	1 (0.9)	3 (2.7)	4 (1.8)	*P*-value for linear trend **0.002**
Normal	17 (15.2)	39 (34.8)	56 (25.0)	
Overweight	43 (38.4)	42 (37.5)	85 (37.9)	
Obese	43 (38.4)	25 (22.3)	68 (30.4)	
Morbid obese	8 (7.1)	3 (2.7)	11 (4.9)	

^*^p-value is significant ≤ 0.05, *SD* standard deviation, *HDL-c* high-density lipoprotein cholesterol, *TG* triglyceride, *FBG* fasting blood glucose, *FIR* fasting insulin resistance, *SBP* systolic blood pressure, *DBP* diastolic blood pressure, *BMI* body mass index

### Number of individual components, categories, and overall prevalence of metabolic syndrome

None of the metabolic syndrome components were present in 1.8% and 15.2% of breast cancer cases and control group, respectively. A single MS component was found in 14.3% of breast cancer patients and 34.8% of control group, two components in 26.8% and 21.4%, and three components in 32.2% and 22.4% of breast cancer and control group, respectively. Four of the components were found in 21.4% and 5.4% and five in 3.6% of breast cancer cases and 0.8% of control group. Table 3 shows a statistically significant linear trend of an increasing proportion of breast cancer cases with an increased number of components, *p* < 0.001. Three components or above which are defined as having metabolic syndrome were found in 57.14% and 28.6% of breast cancer cases and control group, respectively, with an odds ratio of 3.33, 95% CI (1.91–5.81). By categorizing each MS component into high and normal level (according to the Joint Interim Statement (JIS) criteria for the clinical diagnosis of metabolic syndrome in adults) [10], the present study showed that breast cancer patients were more likely to have fasting blood glucose level ≥ 126 mg/dl, systolic and/or diastolic blood pressure ≥ 130 and/or 85 mmHg, triglycerides level ≥ 150 mg/dl, and HDL-C < 50 mg/dl as compared to control group with and all difference were statistically significant (Table 3). Categories of waist circumference were comparable in both study groups with no significant difference (*p* = 0.582).

**Table 3:** Categories and number of individual metabolic syndrome components with its overall prevalence in the two study groups

	Breast cancer *N* = 112 No. (%)	Control *N* = 112 No. (%)	Total *N* = 224 No. (%)	*p*-value *
**FBG**				
< 126 mg/dl	83 (74.1)	102 (91.1)	185 (82.6)	0.001
≥ 126 mg/dl	29 (25.9)	10 (8.9)	39 (17.4)	
**SBP**				
< 130 mmHg	85 (75.9)	100 (89.3)	185 (82.6)	0.008
≥ 130 mmHg	27 (24.1)	12 (10.7)	39 (17.4)	
**DBP**				
< 85 mmHg	89 (76.8)	98 (87.5)	184 (82.1)	0.036
≥ 85 mmHg	26 (23.2)	14 (12.5)	40 (17.9)	
**TG**				
< 150 mg/dl	32 (28.6)	63 (56.2)	95 (42.4)	< 0.001
≥ 150 mg/dl	80 (71.4)	49 (43.8)	129 (57.6)	
**HDL-c**				
< 50 mg/dl	100 (89.2)	66 (58.9)	166 (74.5)	< 0.001
≥ 50 mg/dl	12 (10.8)	46 (41.1)	58 (25.9)	
**WC**				
< 88 cm	54 (48.2)	50 (44.6)	104 (46.4)	0.592
≥ 88 cm	58 (51.8)	62 (55.4)	120 (53.6)	
**Number of components**				
**0**	2 (1.8)	17 (15.2)	19 (8.5)	*P*-value for linear trend < **0.001**
**1**	16 (14.3)	39 (34.8)	55 (24.6)	
**2**	30 (26.8)	24 (21.4)	54 (24.1)	
**3**	36 (32.1)	25 (22.4)	61 (27.2)	
**4**	24 (21.4)	6 (5.4)	30 (13.4)	
**5**	4 (3.6)	1 (0.8)	5 (2.2)	
**Overall prevalence of MS**				
< 3 components	48 (42.9)	80 (71.4)	128 (57.1)	< 0.001
≥ 3 components	64 (57.1)	32 (28.6)	96 (42.9)	

^*^p-value is significant ≤ 0.05, *FBG* fasting blood glucose, *SBP* systolic blood pressure, *DBP* diastolic blood pressure, *TG* triglycerides, *HDL-c* high-density lipoprotein, *WC* waist circumference

Regression analysis results are shown in Table 4 stratified by menopausal status. We found in premenopausal women that an increase of BMI by 1% is met by an increase likely of breast cancer by 40.6%; the presence of family history of breast cancer increased the risk by more than 3 times as those without history, OR, 95% CI (2.97, 1.33–9.66), FBG ≥ 126 mg/dl, and TG ≥ 150 mg/dl at least doubles the risk of breast cancer odds, 95% CI (2.97 (1.06–8.36)) and (2.47 (1.14–5.36)), respectively. An increase in waist circumference by 1 cm is associated with a reduction in risk by 8.6%, OR, 95% CI (0.914 (0.868–0.963) and HDL ≥ 50 mg/dl decreased the risk of breast cancer by 81.3%, OR, 95% CI = 0.187 (0.068–0.513).

**Table 4:** Results of logistic regression for risk of breast cancer stratified by menopausal status

	*B*	S.E	Wald	df	*p*-value	OR	95% CI.for OR	
							Lower	Upper
**Premenopausal (*n* = 166)**								
BMI (%)	0.341	0.083	16.692	1	< 0.001	1.406	1.194	1.656
Waist circumference (cm)	− 0.090	0.027	11.370	1	0.001	0.914	0.868	0.963
Family history of breast cancer	1.276	0.506	6.358	1	0.012	3.583	1.329	9.663
FBG ≥ 126 mg/dl	1.088	0.528	4.247	1	0.039	2.969	1.055	8.357
TG ≥ 150 mg/dl	0.906	0.394	5.280	1	0.022	2.473	1.142	5.355
HDL ≥ 50 mg/dl	− 1.675	0.514	10.606	1	0.001	0.187	0.068	0.513
Constant	− 2.474	1.666	2.206	1	0.138	0.084		
**Postmenopausal (*n* = 58)**								
HDL ≥ 50 mg/dl	− 1.762	0.721	5.969	1	0.015	0.172	0.042	0.706
Constant	2.225	0.899	6.127	1	0.013	9.249		

*p*-value is significant ≤ 0.05, *B* regression coefficient, *SE* standard error of B, *df* degrees of freedom, *OR* odds ratio, *CI* confidence interval, *FBG* fasting blood glucose, *TG* triglycerides, *HDL-c* high-density lipoprotein, *WC* waist circumference

In postmenopausal women, the only component of metabolic syndrome that was associated with breast cancer risk was HDL where HDL level ≥ 50 mg/dl reduces the risk of breast cancer by 82.8%, OR, 95% CI = 0.172 (0.042–0.706).

## Discussion

Metabolic syndrome is a set of medical conditions that include central obesity, high triglyceride, low HDL, hyperglycemia, and hypertension [13]. Recent studies have shown that metabolic syndrome and its related components exert a significant impact on breast cancer risk, development, progression, response to treatment, side effects, and prognosis. These studies attributed it to metabolic abnormalities associated with MS and its components that affect both the general state and organ-specific tumor microenvironment [9, 14].

Since the definition of metabolic syndrome is population specific, the prevalence of metabolic syndrome in breast cancer cases is also population specific. A case–control study conducted in China showed that the prevalence of metabolic syndrome in primarily diagnosed breast cancer patients was significantly higher than control group, 32.6% and 18.2%, respectively [2]. Another cross-sectional study on the East Coast of Peninsular Malaysia showed that the prevalence of metabolic syndrome in breast cancer survivors was 50.5% [15].

Present case–control study in South Egypt Cancer Institute, Assiut University, using the Joint Interim Statement (JIS) criteria for the clinical diagnosis of metabolic syndrome in adults [10], found that the prevalence of metabolic syndrome is higher in the breast cancer patients than control group, 57.1% and 28.6%, respectively, with an odds ratio 3.33, 95% CI (1.91–5.81). Several reasons explain the high prevalence of metabolic syndrome and its components including the low physical activity associated with modernized lifestyle. Additionally, the economic development with changed dietary intake with more fat and calorie consumption also explain the high prevalence. Thus, lifestyle behavior modification with low-fat, low-calorie, appropriate-intensity exercise and weight control would be a good strategy for decreasing metabolic syndrome and its components [2].

This study showed a statistically significant linear trend of an increasing proportion of breast cancer cases with an increased number of metabolic syndrome components. Agnoli et al. suggest that when the number of metabolic syndrome components was considered, the highest number category (≥ 3 components) was associated with significantly greater breast cancer risk, with a significant linear trend [16]. Subjects with individual components < 3 if left with no intervention, or the group of people who were just below the borderline of MS diagnosis, would be more susceptible to have a worse health condition or even being diagnosed with MS in the future [15].

Clinically, metabolic syndrome is defined as the presence of 3 or more of the following factors: high fasting blood glucose, elevated blood pressure, hypertriglyceridemia, reduced high-density lipoprotein cholesterol, and increased waist circumference [3]. Recent studies suggest alarming association between hyperglycemia and various cancers as high glucose levels inhibit apoptosis resulting in increased cell viability under hypoxic conditions, thereby facilitating cell survival and malignant progression [17]. The current study showed that the prevalence of high fasting blood glucose is statistically higher in breast cancer cases (25.9%) than the control group (8.9%), and in premenopausal women, the risk of breast cancer was more than double compared to the control group, adjusted odds ratio 2.97, 95% CI, (1.1–8.4). A similar study in Mexico showed that women with prediabetes and diabetes were more vulnerable to breast cancer risk; the adjusted odds ratio for prediabetics was 2.08, 95% CI 1.10–3.96, and for diabetics, 2.85, 95% CI 1.55–5.26 [18]. As well, diabetic patients were found to have a slight chance of later stage at presentation of breast cancer as well as poorer outcome in comparison with the non-diabetic patients in a hospital record-based descriptive cross-sectional study conducted in India [19]. Women with hypertension have common risk factors with breast cancer including overweight and obesity, physical inactivity, and poor dietary habits [20]. The present study showed a higher prevalence of hypertension in breast cancer cases than in the control group 24.1% versus 8.9%, respectively. Similar results were found in a study by Alsolami et al. (2019), where hypertension was found to be more frequent in breast cancer patients when compared to the control group (48.6% vs. 15.1%, respectively), in a study in Makkah, Saudi Arabia, searching for determinants of breast cancer [21]. Dyslipidemia also has been associated with an increased risk for developing cancer such as breast cancer growth and metastasis. Studies found that dyslipidemia affects mammary tumor growth and metastasis because of a protein called apolipoprotein E (ApoE) glycoprotein, a protein that functions as a regulator of plasma lipid levels [22]. Uen et al. demonstrated that the posttranslational modifications of ApoE play roles in tumor development [23]. Also, Xu et al. reported that serum ApoE levels are notably upregulated in patients with breast cancer [24]. For hyperlipidemia, we found that breast cancer patients were more likely to have higher total cholesterol levels, higher triglycerides levels (adjusted OR in premenopausals was 2.47 (1.14–5.36), lower HDL-c level (adjusted OR in pre and postmenopausal women were 0.187 (0.068–0.513) and 0.172 (0.042–0.706), respectively. Hyperlipidemia was also found with a higher prevalence in breast cancer cases when compared to women with benign breast disease and normal women in Bangladesh [25].

While BMI was associated with increased risk in premenopausal women, waist circumference (cm) was comparable among cases and controls in univariate analysis, and with logistic regression analysis, we found that an increase in waist circumference of premenopausal women by 1 cm was associated with a reduction in risk by 8.6%, OR, 95% CI (0.914 (0.868–0.963). This finding could be explained by the late diagnosis of breast cancer patients especially with the high level of illiteracy and the associated anxiety and depression leading to anorexia and rapid weight loss. There is a wide range of variation in the results of the associations between waist circumference and breast cancer risk. Some studies showed that waist circumference was not associated with breast cancer risk. Chen et al. (2016) found that it was not associated with premenopausal breast cancer [26]. Other studies, like the present study, showed an inverse association between waist circumference and breast cancer risk. In a multicentric population-based case–control study, which investigated the associations between excess adiposity, body shape evolution across life, and risk of premenopausal breast cancer, negative associations between adult adiposity and breast cancer risk were observed in weight, waist circumference, and BMI [27]. Similar findings were found in a population-based case–control study in South African black women [28]. Other studies supported the positive association of high waist circumference and breast cancer risk. The interpretation of such opposite associations between different measures of adiposity remains complex. In particular, waist circumference is only a proxy for body fat distribution, and techniques such as DEXA (dual-energy X-ray absorptiometry) or impedance, which provide information on body composition and fat distribution, would be helpful in disentangling these associations [27].

To the best of our knowledge, the present study is the first to evaluate the association between metabolic syndrome and breast cancer risk in Upper Egypt. The study strictly followed the diagnostic criteria of MS rather than neglecting or replacing components, and the criteria used for MS definition were relatively recent. Therefore, due to the rarity of research tackling this point, the present study adds to the knowledge for future research in Egypt and the surrounding region. The present study was an observational case–control one, so a prospective design may be beneficial in assessing weight gain, its timing, and its effect on breast cancer risk and prognosis. We should also take into consideration that we involved female population with specific demographic, socioeconomic, cultural/behavioral, and anthropometric characteristics that may be different from those of other populations living in other areas inside and outside Egypt. Therefore, caution is necessary before generalization of results of the present study in other contexts.

## Conclusions

In conclusion, metabolic syndrome and its components are present to a greater degree in breast cancer women compared to the control population and the risk of breast cancer increased as the number of MS components increased.

Individual metabolic syndrome components are associated also with the risk of breast cancer especially, the low level of HDL, high level of triglycerides, and high fasting blood glucose level. We believe that prevention or reversal of metabolic syndrome by raising community awareness for lifestyle changes could be an effective way in minimizing the toll of the disease.

## Data Availability

Detailed data is in the tables.

## Abbreviations

ApoE: Apolipoprotein E
BMI: Body mass index
CI: Confidence interval
DBP: Diastolic blood pressure
DEXA: Dual-energy X-ray absorptiometry
Df: Degrees of freedom
FBG: Fasting blood glucose
FIR: Fasting insulin resistance
HDL: High-density lipoprotein
HRT: Hormonal replacement therapy
JIS: Joint Interim Statement
MS: Metabolic syndrome
NCEP ATP III: National Cholesterol Education Program Adult Treatment Panel III
OCP: Oral contraceptive pills
OR: Odds ratio
SBP: Systolic blood pressure
SD: Standard deviation
SE: Standard error
SECI: South Egypt Cancer Institute
TG: Triglycerides
WC: Waist circumference
